# Robotic sleeve resection for pulmonary disease

**DOI:** 10.1186/s12957-018-1374-x

**Published:** 2018-04-02

**Authors:** Chengqiang Li, Bin Zhou, Yu Han, Runsen Jin, Jie Xiang, Hecheng Li

**Affiliations:** Department of Thoracic Surgery, Ruijin Hospital, Shanghai Jiaotong University School of Medicine, 197 Ruijin 2nd Road, Shanghai, 200025 China

**Keywords:** Robotic sleeve resection, Suture mode, Lung cancer, Lung parenchyma preserving, Segmental bronchial sleeve

## Abstract

**Background:**

Few studies have described robotic sleeve resection with pulmonary resection. Here, we report the successful implementation of a completely portal robotic sleeve resection with or without pulmonary resection using a modified suture mode.

**Methods:**

In total, 339 patients underwent curative robotic pulmonary surgery at Ruijin Hospital between May 2015 and September 2017. Three of these patients underwent robotic sleeve resection (right upper lobe, one; left upper lobe, one; and lingular segmental bronchus, one). Five port incisions were utilized, and a simple continuous running suture combined with two interrupted sutures of the membranous and cartilaginous junction portion was preferred for the anastomosis.

**Results:**

The postoperative course was uneventful for two patients with squamous cell carcinoma. The lingular segmental bronchus patient without pulmonary resection (a salivary gland tumor) underwent short-term atelectasis. The median operation time was 155 (range 132–230) minutes. The median anastomosis time was 25 (range 23–32) minutes. The median length of postoperative hospital stay was 7 (range 6–10) days. There was no mortality or conversion to thoracotomy for any of the patients. All patients were followed for 3–6 months, and there is no tumour recurrence.

**Conclusions:**

Our limited experience suggested that robotic sleeve resection for pulmonary disease with or without pulmonary resection may be safe and effective. The anastomosis time can be shortened with more robotic surgery experiences and the modified suture mode.

## Introduction

The first published bronchial sleeve resection was performed in 1947 [1]. Sleeve resection is a better choice than pneumonectomy when operating on central lung cancer and low-grade neoplasms because of its advantages in terms of improved morbidity, mortality, and lung function preservation [2–7], even after induction therapy [8–11]. The key part of sleeve resection is reconstruction, and this remains challenging even during thoracotomy. However, like the other thoracic surgeries, sleeve resection has continuously evolved in the era of video-assisted thoracoscopic surgery [12]; however, due to its technical difficulty, this procedure has been adopted slowly. Surgical robotics might represent a viable solution to this technically complex procedure. In this article, to our knowledge, we describe all the literature published in English on robotic sleeve lobectomy in patients (Table 1) and present our experience, focusing on the suture mode of the operation.

**Table 1:** Literature data

Author	*N*	Operation time (min)	Bleeding (ml)	Suture mode	Chest tube stay (days)	Postoperative hospital stay (days)	Morbidity	Mortality	3 months recurrence
Schmid et al. 2011 [26]	1	364	–	Interrupted and running	9	15	0	0	0
Nakamura et al. 2013 [27]	1	403	170	Interrupted	2	–	0	0	0
Pan et al. 2015 [28]	1	245	200	Running	5	10	0	0	0
Cerfolio 2015 [29]	8	–	–	Interrupted and running	–	–	AF 1	0	0
Zhao et al. 2016 [30]	1	–	100	Running	3	7	0	0	0
Lin et al. 2016 [31]	6	436.7 ± 200.2^#^	750 ± 1005^#^	Running	5.3 ± 4.5^#^	11.3 ± 9.1^#^	Stenosis 1 pneumonia 1	0	1
Pan et al. 2016 [32]	21	158.4 ± 42.0^#^	157.1 ± 97.8^#^	Running	9.0 ± 8.2^#^	10.7 ± 7.6^#^	19%*	1	0
Qiu et al. 2016 [33]	1	240	150	Running	3	6	0	0	0

*N* number, *AF* atrial fibrillation

^#^Data are presented as the mean ± SD

*Data are presented as *n* (%)

## Material and methods

### Patient demographics

From May 2015 until September 2017, 339 patients underwent curative robotic pulmonary surgery in our department; 236 patients underwent robotic lobectomy, 78 underwent segmentectomy, 22 underwent wedge resection, and 3 underwent sleeve resection. Of the three sleeve resection cases, there were two cases of sleeve lobectomy with bronchoplasty and one case of lingular segmental bronchial sleeve resection without pulmonary resection.

All three robotic sleeve patients were male. Routine laboratory blood tests, electrocardiographic examination, and lung function tests were performed to evaluate the feasibility of robotic sleeve resection. Preoperative ultrasonography of superficial lymph nodes (cervical and supraclavicular lymph nodes), brain magnetic resonance imaging, enhanced abdominal computed tomography (CT), bone scanning, and whole-body positron emission tomography-computed tomography scanning were used to exclude metastases. The tumour location and pathology were evaluated by enhanced chest CT and electronic bronchoscopy. Endo-bronchial ultrasound-guided transbronchial needle aspiration or mediastinoscopy was performed to exclude N2 disease. Two patients had squamous cell carcinoma (one each located in the right and left hilum), and the other had a salivary gland tumour located in the lingular segmental bronchus. The patient with a left upper lobe tumour received 2 cycles of neo-adjuvant chemotherapy (cisplatin 75 mg/m^2^ on day 1 plus gemcitabine 1.25 g/m^2^ on day 1 and on day 8; 3 weeks per cycle). Three weeks after induction therapy, the clinical restage was stable; then, the patient was proposed for a robotic thoracic surgical procedure. Pathological staging was based on the eighth edition of the International Association for the Study of Lung Cancer guidelines (Table 2).

**Table 2:** Demographic and preoperative variables

*N*	Age (years)	Sex	Symptoms	FEV1 (L)	FEV1 (%)	Tumour location	Histologic type	
Case 1	71	Male	Cough	2.62	83.1	RUL	SCC	
Case 2	53	Male	Cough	1.41	53.5	LUL	SCC	
Case 3	29	Male	None	3.97	96.6	LLS	Salivary gland tumour	

*N* number, *FEV1* forced expiratory volume in 1 sec, *RUL* right upper lobe, *LUL* left upper lobe, *LLS* left lingular segment, *SCC* squamous cell carcinoma

### Surgical procedure

**Fig. 1:**
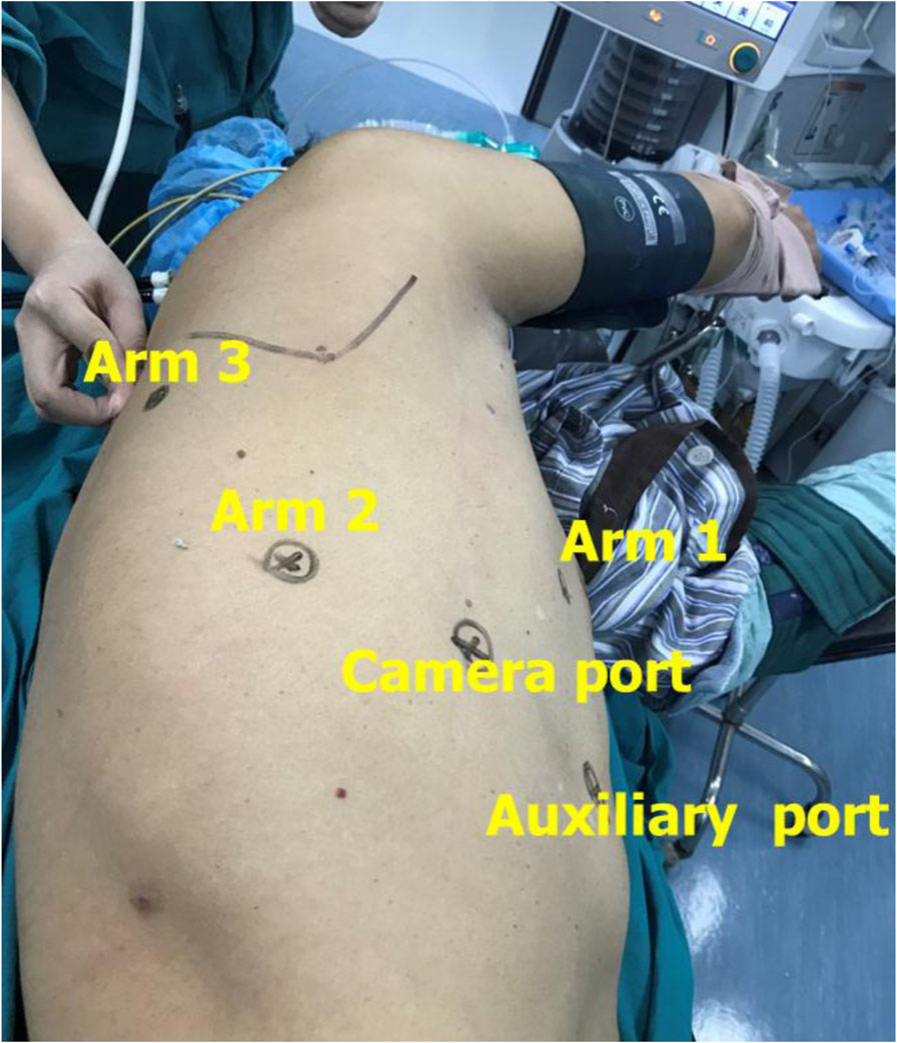
Schaematic diagram of patient position and incision location. Arm 1, fifth ICS at the anterior axillary line; arm 2, eighth ICS at the posterior axillary line; arm 3, eighth ICS, 2 cm from the spine; camera port, eighth ICS at the middle axillary line; an auxiliary port, the eighth ICS near the costal arch. ICS, intercostal space

After the induction of general anaesthesia, the patient was placed in a left or right lateral decubitus position with double lumen endotracheal intubation. We prefer completely portal robotic surgery using the da Vinci Si surgical robot (Intuitive Surgical, Inc., Santa Clara, CA, USA). The camera port was created in the eighth intercostal space (ICS) of the middle axillary line. The working port for arm 1 was on the fifth ICS of the anterior axillary line, and the remaining three ports were all on the eighth ICS (arm 2 at the posterior axillary line, arm 3 at 2 cm from the spine and the 8-mm auxiliary port near the costal arch) (Fig. 1). The robot patient cart was positioned directly above the operating table. A unipolar cautery hook was used in the arm 1. The arm 2 was connected with bipolar cautery grab. The arm 3 was used to track the lung at the discretion of the surgeon.

**Fig. 2:**
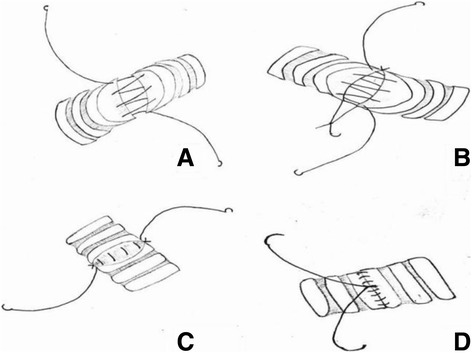
Schaematic diagram for sleeve bronchoplasty. **a** Continuous suture of the membranous part from posterior to anterior with a two-armed 3-0 Prolene. **b** Two interrupted sutures at the membranous and cartilaginous junction portions. **c** The two-armed 3-0 Prolene was tightened and tied with the former interrupted suture at each side. **d** Simple running suturing of the cartilaginous part

For two patients with squamous cell carcinoma, on entering the thoracic cavity, warmed humidified CO_2_ was insufflated in the chest to maintain a pressure of 10 mmHg. The thoracic cavity was explored to confirm the absence of metastasis and to decide whether sleeve resection was feasible. The inferior pulmonary ligament was divided to reduce tension during and after the anastomosis. The no. 9 lymph node was retrieved, and then en bloc no. 7 lymph node resection was performed. The posterior mediastinum pleura was then opened, and the no. 4L lymph node was dissected for the left-sided case. Robotic arm 3 was then used to retract the lung posteriorly while robotic arms 1 and 2 were used to open the anterior mediastinum pleura and remove the no. 2R and 4R lymph nodes for the right-sided case and the nos. 5 and 6 nodes for the left-sided case. The lobar vein and arteries were dissociated, and the vein, fissure, and pulmonary arteries were subsequently resected using the endoscopic linear stapler. The bronchi were then divided and transected using electric scissors. The specimen was temporarily placed in the diaphragmatic sinus. We performed end-to-end bronchial anastomosis using a simple running suture combined with two interrupted sutures of the membranous and cartilaginous junction parts. A double-armed 3-0 Prolene suture was used. The continuous suture of the membranous part was completed from posterior to anterior with one needle, keeping the line loose (Fig. 2a). Then, one interrupted suture was completed at each of the membranous and cartilaginous junction portions. The knots were tied to pull the proximal and distal bronchial stumps together (Fig. 2b); the double-armed 3-0 Prolene suture was tightened and tied with the former interrupted suture at each side (Fig. 2c). The remaining cartilage parts were sutured with a double-armed Prolene suture, and the last knot was tied on the middle of the anterior portion with careful regulation of the suture tightness (Fig. 2d).

**Fig. 3:**
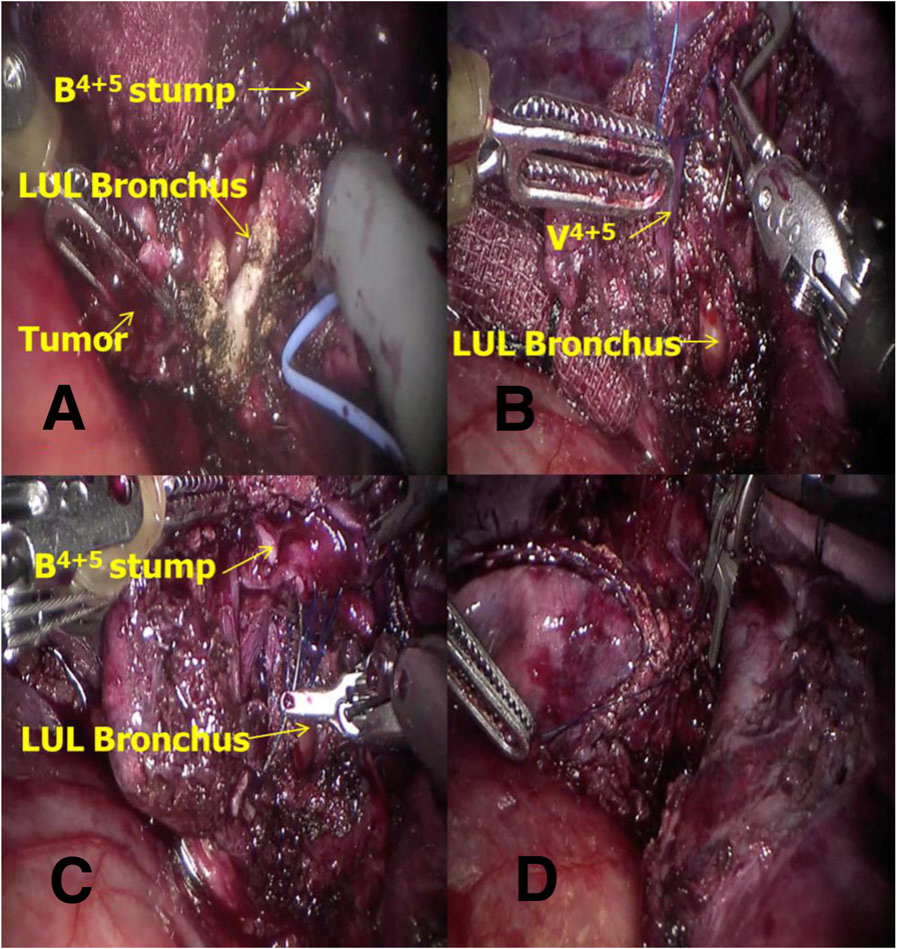
Schaematic diagram of lingular segment bronchial sleeve resection. **a** Cut open the bronchus and a round tumour with intact membrane was then revealed. **b** Narrow the rim of LUL bronchus to better match the calibre of the distal segment bronchus with a 5-0 Prolene. **c** End-to-side bronchial anastomosis was performed by a 5-0 PDS II continuous running suture. **d** The knot was placed on the anterior portion of the bronchus

Regarding the lingular segmental bronchial sleeve resection without pulmonary resection, the arteries and veins were all preserved. The lingular segmental bronchus was exposed. Electrical scissors were used to cut open the lingular bronchus (Fig. 3a). The negativity of the bronchial stumps was confirmed by frozen pathological examination before bronchial anastomosis. The side of the upper segment of the trachea was narrowed to better match the calibre of the distal segmental bronchus with 5-0 Prolene (Fig. 3b). There was no tension between the two sides; therefore, an end-to-side bronchial anastomosis was performed using a 5-0 polydioxanone synthetic absorbable suture (PDS II) with continuous running sewing (Fig. 3c, d).

After these above procedures, one of the ports was enlarged, and the specimen was placed into an Endobag and retrieved through it. The lung was inflated with 30 cm of H_2_O airway pressure under saline to ensure that there were no air leaks. A 20-F chest tube was placed in the eighth ICS.

## Results

The operative and postoperative variables were showed in Table 3. There were no mortalities or serious complications. The operative time was from incision to closure. The chest tube was removed once there was no air leakage and there was less than 200 ml of pleural fluid per day. One patient underwent a short-term atelectasis of the anastomosed lingular segment. After bronchoscope sputum suction and antibiotic treatment, the patient was discharged on postoperative day 10. Fortunately, the lingular segment achieved full re-expansion during follow-up.

**Table 3:** Operative and postoperative variables

*N*	OT (min)	AT (min)	Bleeding	Postoperative hospital stay (days)	Chest tube stay (days)	Pathological stage	Complication	Mortality	3 months recurrence
Case 1	132	23	100	6	4	T2aN0M0	None	None	None
Case 2	230	25	150	7	5	T2bN1M0	None	None	None
Case 3	155	32	75	10	3	T1bN0M0	Atelectasis	None	None

*N* number, *OT* operative time, *AT* anastomotic time

## Discussion

The first study describing video-assisted thoracoscopic (VATS) sleeve lobectomy was reported in 2002 [13]; after more than 10 years, there have been only a small number of case series reports [14–18], although the morbidity, mortality, and survival were comparable between VATS sleeve lobectomy and thoracotomy. The slow adoption of the thoracoscopic technique for sleeve resection is mainly due to the technical challenges involved in the bronchial anastomosis in VATS and its steep learning curve [19].

With the advent of modern technology, surgical robotics has come of age. Robotic-assisted thoracic surgery has many advantages over conventional VATS, such as an additional four degrees of freedom, superior 3-D vision from the binocular camera, tremor filtration, elimination of the fulcrum effect, and improved ergonomic positioning for the surgeon [20–22]. These advantages facilitate procedures that are typically difficult in conventional VATS, such as suturing and knot tying, and surgeons, including residents, have demonstrated significantly better suturing and knot-tying capabilities using the robotic surgical system [23–25].

The first clinical case of robotic sleeve lobectomy was reported by Doctor Schmid in 2011 [26]. The airway reconstruction was performed using the da Vinci robot. It takes 50 min to accomplish the anastomosis, and the da Vinci robot facilitates the technically challenging procedure. Subsequently, seven reports of robot-assisted sleeve lobectomy have been published in the English literature. According to these publications, the performance of the bronchial anastomosis varied according to the surgeons’ preferences (Table 1).

Our own results, in terms of short-term outcomes, operation time, and morbidity rate, are comparable to those reported in the literature [26–33]. The median bronchial anastomosis time was 25 min. Schmid and coworkers [26] reported an anastomosis time of 50 min. Nakamura and associates [27] reported an anastomosis with 16 stitches of interrupted sutures that required a long time because the thread was loosened or cut while ligating. In VATS sleeve lobectomy, Chen and colleagues reported a mean anastomosis time of 37.6 ± 12.0 min [18], and Wang and associates reported a median time of 44 (37–48) minutes for bronchial anastomosis [15]. The main features of our experience are the suture mode and segment bronchial sleeve resection without pulmonary resection.

In our practice, end-to-end bronchial anastomosis using a simple running suture combined with two interrupted sutures of the membranous and cartilaginous junction portions was preferred. In our experience, there are several advantages to using this suture mode. First, we did not put the proximal and distal bronchial stumps together at the beginning of the suture; this not only made the continuous suture of the membranous part easier with double-armed 3-0 Prolene sutures but also minimized the possibility of clamp injury. Second, the two interrupted sutures were tied to pull the proximal and distal bronchial stumps together easily, and this use of two interrupted sutures prevented potential lateral air leaks. Third, the double-armed 3-0 Prolene was tightened without tension and was then tied with the former interrupted suture at each side; this facilitated the running suture of the remaining cartilaginous parts.

We reported the first case of robot-assisted lingular segmental bronchial sleeve resection while totally preserving the lung parenchyma. Segmental bronchial sleeve resection is complex, and the complication rate is possibly increased compared to the standard sleeve resection [34]. Indeed, the patient who underwent lingular segment bronchial sleeve resection experienced a short-term atelectasis. As a result of the therapeutic interventions, the lingular segment achieved full re-expansion during follow-up.

Despite the advantages of robotic sleeve resection have been reported [26–33], the technique also has some potential drawbacks. First, robotic sleeve resection requires four to five incisions, but VATS only requires two to three incisions, sometimes even only a single port. This may increase the postoperative pain and decrease the aesthetic outcome. Second, the cost is higher; in our centre, an extra 30,000 RMB must be paid for the robotic surgery. Third, the setup of the robotic system is time-consuming. Finally, the worst disadvantage of robotic surgery is the inability of the surgeon to use the tactile sense. We have performed more than 500 robotic surgeries with a single surgeon and the same team. The setup time was reduced as the team experience increased, and the surgeon was able to partially compensate for the lack of haptic feedback by visually observing the deformation of tissue while suturing and knot tying. Some researchers and engineers are working on a means of relaying haptic feedback directly to the surgeon’s control actuators [24, 35].

## Conclusion

Our limited experience demonstrates that robotic sleeve resection with or without pulmonary resection appears safe and feasible. The anastomosis time can be shortened with an increasing number of robotic surgery experiences and a modified suture mode.
